# DNA2 acts as a brake on β cell insulin hypersecretion and diet-induced metabolic dysfunction

**DOI:** 10.3389/fcell.2026.1733190

**Published:** 2026-03-04

**Authors:** Haixia Xu, Dongmei Tang, Na Yang, Meilin Ma, Yan Tian, Xianghui Fu

**Affiliations:** 1 State Key Laboratory of Biotherapy and Cancer Center, Department of Pharmacy, Institute of Metabolic Diseases and Pharmacotherapy, West China Hospital, Sichuan University, Chengdu, Sichuan, China; 2 State Key Laboratory of Biotherapy and Cancer Center, Department of Biotherapy, Center for Diabetes and Metabolism Research, West China Hospital, Sichuan University, Chengdu, Sichuan, China

**Keywords:** DNA2, hyperinsulinemia, insulin secretion, mitochondrial activity, β cell proliferation

## Abstract

**Purpose:**

DNA replication helicase/nuclease 2 (DNA2) is an evolutionarily conserved nuclease-helicase with known role in maintaining nuclear genome stability. However, its potential involvement in metabolic regulation and disease remains unclear. This study investigates the role of DNA2 in pancreatic β cell physiology and diabetes pathogenesis.

**Methods:**

β cell-specific DNA2 knockout mice (DNA2^INS2−/−^) were generated and fed either a chow diet (CD) or high-fat diet (HFD). Metabolic phenotyping, insulin secretion assays, transcriptomic profiling, mitochondrial function analysis, and ultrastructural imaging were performed. INS-1 cells were used to assess the functions of DNA2 *in vitro* through knockdown, overexpression and site-directed mutagenesis.

**Results:**

DNA2^INS2−/−^ mice exhibited normal metabolic profiles under CD, but developed severe hyperglycemia, hyperinsulinemia, insulin resistance, and ectopic lipid deposition upon HFD feeding. This phenotype was accompanied by increased β cell proliferation and glucose-stimulated insulin secretion. RNA sequencing revealed the dysregulation of mitochondrial regulatory genes in DNA2-deficient islets. Functional assays confirmed that DNA2 deletion enhanced mitochondrial ATP production and oxidative phosphorylation, whereas its overexpression suppressed mitochondrial activity. Domain-specific mutagenesis demonstrated that both nuclease and helicase activities are essential for DNA2-mediated metabolic regulation.

**Conclusion:**

Our findings identify DNA2 as a negative regulator of mitochondrial bioenergetics and insulin secretion in β cells. By limiting mitochondrial activity, DNA2 serves as a rheostat that prevents β cell overactivation during metabolic stress, thereby preserving systemic glucose homeostasis.

## Highlights

- DNA2 acts as a β-cell rheostat limiting mitochondrial energy production
- Loss of DNA2 drives β-cell proliferation and hyperinsulinemia under HFD
- DNA2 deficiency enhances mitochondrial ATP production and insulin secretion
- Both nuclease and helicase domains are essential for the regulatory role of DNA2

## 1 Introduction

Type 2 diabetes (T2D) represents a worldwide metabolic epidemic, distinguished by persistent hyperglycemia due to insulin resistance and the progressive deterioration of β cell activity (Accili et al., 2025; Li et al., 2024; Cao et al., 2023). In addition to impaired glycemic control, T2D significantly increases the risk of cardiovascular complications, neuropathies, nephropathies, and certain cancers (Xie et al., 2025; Ma et al., 2025; Savelieff et al., 2025; Liao et al., 2025). Although lifestyle modifications and pharmacological interventions, such as metformin and insulin therapy are widely used, current interventions often fail to achieve sustained glycemic control and prevent long-term complications (Crawford and Laiteerapong, 2024; Tang et al., 2025; Tang et al., 2023). Beyond diabetes, chronic overnutrition and obesogenic environments profoundly disrupt systemic metabolic homeostasis, promoting insulin resistance and ectopic lipid accumulation (Jin et al., 2023). In the liver, these disturbances contribute to metabolic dysfunction–associated steatotic liver disease (MASLD), which is increasingly recognized as a multisystem metabolic condition associated with diet-related metabolic stress and systemic metabolic dysfunction (Henin et al., 2024).

Pancreatic β cells are the sole producers of insulin, the primary hormone responsible for lowering blood glucose levels (Pinheiro-Machado et al., 2020; Mezza et al., 2019; Roden and Shulman, 2019). In response to metabolic stress, such as insulin resistance and overnutrition, β cells undergo functional and structural adaptations, including increased insulin secretion and β cell mass expansion (Roden and Shulman, 2019). Although initially compensatory, this response can progress to hyperinsulinemia that is now recognized as a consequence and contributor to insulin resistance and metabolic deterioration (Mezza et al., 2019; Roden and Shulman, 2019; Arai et al., 2025). Growing evidence indicates that targeting β cell hyperactivity and insulin overproduction may offer novel avenues to alleviate metabolic stress (Hill, 2022; Ghasemi Gojani et al., 2024). Therefore, elucidating the molecular mechanisms underlying β cell adaptation and failure is crucial for identifying novel therapeutic strategies for T2D and its complications.

Compensatory β cell hyperplasia is primarily driven by increased β cell replication and regulated by primary signaling pathways, including forkhead box O transcription factor 1 (FoxO1), insulin receptor substrate 2 (Irs2), and AKT serine/threonine kinase (Akt1) (Okamoto et al., 2006; Terauchi et al., 2007; Shirakawa, 2023). Similarly, glucose-stimulated insulin secretion (GSIS) relies heavily on mitochondrial metabolism (Jezek et al., 2022; Rovira-Llopis et al., 2017). ATP generated through mitochondrial oxidative phosphorylation serves as a crucial signal that associates glucose metabolism with insulin granule exocytosis, making mitochondrial integrity and function essential for sustained insulin secretion (Jacovetti et al., 2024; Jaburek et al., 2024), although the underlying mechanisms remain unclear.

DNA replication helicase/nuclease 2 (DNA2) is a helicase-nuclease implicated in DNA repair and genome maintenance (Falkenberg et al., 2024). Pathogenic variants in DNA2, although rare, have been associated with multisystemic disorders, including progressive myopathy, sensorineural hearing loss, Seckel syndrome, and congenital myopathy (Mudassir and Agha, 2023; Sun et al., 2022; Gonzalez-Del Angel et al., 2019; Ronchi et al., 2013). Despite these well-documented roles (Falquet et al., 2020; Paudyal et al., 2017; Zhai et al., 2023), the function of DNA2 in pancreatic β cell biology and systemic metabolic regulation remains unexplored.

In this study, we generated pancreatic β cell-specific DNA2-knockout mice to assess the role of DNA2 in systemic metabolic homeostasis and β cell function. Under physiological conditions, β cell-restricted DNA2 deletion had minimal effects on whole-body metabolism but enhanced GSIS, resulting in increased circulating insulin levels. Under high-fat diet (HFD) challenge, DNA2 deficiency significantly exacerbated obesity, hepatic steatosis, systemic insulin resistance, and compensatory hyperinsulinemia. Mechanistically, β cell DNA2 loss triggered islet hyperplasia through enhanced β cell proliferation and increased insulin secretion. Integrative transcriptomic and functional profiling demonstrated that DNA2 loss triggers mitochondrial hypertrophy and hyperactivation in β cells, driving excessive ATP synthesis and insulin production. These findings reveal a previously unrecognized regulatory axis in which DNA2 governs β cell mitochondrial dynamics and highlight its pathophysiological relevance in obesity-associated insulin resistance and T2D.

## 2 Materials and methods

### 2.1 Animals

DNA2 flox/flox mice (DNA2^fl/fl^) were kindly provided by Professor Weiqiang Lin (Zhejiang University). β cell-specific DNA2 knockout (DNA2^INS2−/−^) mice were generated by crossing DNA2^fl/fl^ mice with Ins2-Cre mice (Stock No. 003573). The knockout strategy is shown in Supplementary Figure S1A, and genotyping was performed by PCR on tail and islet DNA. Age-matched littermates were used in all experiments. Mice were housed under specific pathogen-free conditions with controlled temperature, humidity, a 12-h light/dark cycle, and *ad libitum* access to chow and water. Male DNA2^INS2−/−^ and DNA2^fl/fl^ littermates were 6–8 weeks of age at the initiation of the experimental protocol. Age- and weight-matched male DNA2^INS2−/−^ and DNA2^fl/fl^ littermates were fed CD (#1025; Beijing Hua Fu Kang) or HFD (60% kcal fat, #D12492; Research Diets) for ≥16 weeks to establish diet-induced obesity. Body weight was measured weekly throughout the experimental period. Food intake was assessed during weeks 13–16 of HFD feeding by measuring weekly food consumption per cage. All procedures were approved by the Institutional Animal Care and Use Committee of Sichuan University.

### 2.2 Genotyping

Genomic DNA was extracted from the mouse tail or islet biopsies for genotyping. The presence of the Dna2 floxed allele was determined by PCR using the following primers: 5′-AGT​TTT​AGC​TAG​CCG​GGA​ACT​C-3′ (Dna2_F2), 5′-CGC​TGC​CTT​AGT​TCT​TTG​GAT​A-3′ (Dna2_R2) (512 bp, WT; 625 bp, flox, Neo-deleted). The presence of the Ins2-Cre transgene was determined by PCR using the following primers: 5′-CAT​ATT​GGC​AGA​ACG​AAA​ACG​C-3′ (Cre_F); 5′-CCT​GTT​TCA​CTA​TCC​AGG​TTA​CGG-3′ (Cre_R) (413 bp, Cre positive). The identification of DNA2^INS2−/−^ mice was determined by PCR using the following primers: 5′-CTA​ATG​CGG​GAT​CCA​GGA​CTC​AAA​C-3’ (Dna2_F1), 5′-CGC​TGC​CTT​AGT​TCT​TTG​GAT​A-3’ (Dna2_R2) (210 bp).

### 2.3 Cell culture

INS-1 cells (the gift from Prof. Chunbo Teng, Northeast Forestry University) were maintained in RPMI 1640 supplemented with 10% FBS, 1 mM sodium pyruvate, 2 mM glutamine, 50 μM β-mercaptoethanol, 10 mM HEPES, and 1% P/S. Min6 cells were grown in RPMI 1640 containing 10% FBS, 2% B27, 2 mM glutamine, 50 μM β-mercaptoethanol, and 1% P/S. 293T cells (ATCC CRL3216) were cultured in DMEM with 10% FBS and 1% P/S.

For siRNA knockdown, cells were transfected with 50 nM siRNA using HiPerFect (#301705; QIAGEN). Cells were harvested 24 h after transfection for gene expression analysis and 48 h after transfection for functional assays. Two independent siRNAs targeting murine DNA2 (mDNA2) (5′-ATG​GCA​GGT​GAC​AGG​ATT​ATT-3′ and 5′-ACG​CTG​GAG​TCG​CAA​TCT​AAA-3′) and a non-targeting control (5′-UUC​UCC​GAA​CGU​GUC​ACG​UTT-3′) were used.

### 2.4 Plasmids and transfection

Murine DNA2 cDNA was cloned into pcDNA3.1 (mDNA2^WT^). Point mutants (mDNA2^D278A^, mDNA2^K655E^, and mDNA2^D278A+K655E^) were generated by site-directed mutagenesis (primers in Supplementary Table S1). Plasmids were transfected using Attractene (#1051563; QIAGEN) following the manufacturer’s instructions.

### 2.5 Islet isolation and culture

Islets were isolated by collagenase XI (#C7657; Sigma) digestion as previously described (Xu et al., 2020) with slight modifications. Hand-picked islets were cultured in RPMI 1640 supplemented with 10% FBS.

### 2.6 Blood glucose and insulin measurement

Blood glucose and insulin were measured as described (Xu et al., 2020). Random glucose was assessed in fed mice; fasting glucose after 16 h fasting. Glucose was measured using a portable glucometer (Abbott Laboratories). Serum insulin was quantified with the Ultra-Sensitive Mouse Insulin ELISA Kit (#90080; Crystal Chem). The formula for calculating the homeostasis model assessment of insulin resistance (HOMA-IR) index, is fasting blood glucose (mmol/L) multiplied by fasting blood insulin (μU/mL) and then divided by 22.5.

### 2.7 Glucose tolerance test (GTT) and insulin tolerance test (ITT)

For GTT, mice were fasted for 16 h, injected intraperitoneally with D-glucose (#G7021; Sigma), and glucose levels measured at 0, 15, 30, 60, 90, and 120 min. For ITT, mice were fasted for 6 h, injected with human insulin (#HI0219; Lilly), and glucose recorded at 0, 15, 30, 60, and 90 min (Xu et al., 2019).

### 2.8 Immunofluorescence (IF) and immunohistochemistry (IHC) staining

Paraffin-embedded pancreatic sections were deparaffinized, rehydrated, and subjected to antigen retrieval in 10 mM Tris-EDTA (pH 9.0). Sections were blocked with PBS containing 1% BSA and 5% goat serum. For IF, anti-Insulin antibody (#ab181547, Abcam) and Alexa Fluor® 488 Anti-Glucagon antibody (#ab307340, Abcam) were applied overnight at 4 °C, followed by Alexa Fluor 594-conjugated goat anti-rabbit IgG (#A-11012, Invitrogen). Images were captured on a confocal microscope (LSM 880; Zeiss). For IHC, sections were incubated with anti-insulin antibody (#ab181547, Abcam), followed by HRP-conjugated secondary antibody (#31460, Invitrogen) and DAB substrate (#34002, Invitrogen), then counterstained with hematoxylin. ImageJ (v1.54) was used for analysis.

### 2.9 RNA extraction and quantitative reverse transcription polymerase chain reaction (qRT-PCR)

Pancreatic islets were hand-picked after collagenase digestion and immediately preserved in RNAlater (#76104; Qiagen) before RNA isolation with TRIzol (#15596026; Thermo Fisher). cDNA was synthesized using M-MLV Reverse Transcriptase (#28025013; Invitrogen). qRT-PCR was performed in 10 μL reactions with SYBR Green Master Mix (#204145; Qiagen) and specific primers (listed in Supplementary Table S2).

### 2.10 *In vitro* insulin release from islets

Hand-picked mouse islets were cultured overnight and preincubated for 30 min in Krebs buffer containing 2.8 mM glucose, followed by stimulation with 2.8 mM glucose in the presence or absence of secretagogues (16.7 mM glucose, 20 mM arginine, or 30 mM KCl) for 1 h. Culture supernatants were collected for insulin secretion analysis. Total insulin content was extracted from the same islets using acid–ethanol solution. Insulin concentrations were measured using an Ultra-Sensitive Mouse Insulin ELISA Kit (#90080; Crystal Chem) according to the manufacturer’s instructions, and insulin secretion was normalized to total insulin content.

### 2.11 Oxygen consumption assay

INS-1 cells (10,000 cells/well) were seeded in XFp plates, transfected for 48 h, and incubated 1 h in assay medium (16.7 mM glucose, 2 mM glutamine, 1 mM pyruvate). Oxygen consumption was measured with the XF Extracellular Flux Analyzer (Seahorse), with sequential injections of oligomycin (4 μM), FCCP (3 μM), antimycin A (0.5 μM), and rotenone (0.5 μM).

### 2.12 Hematoxylin and eosin (H&E) staining

Fresh liver, epididymal white adipose tissue (eWAT), brown adipose tissue (BAT), and pancreas were fixed in 10% formalin, paraffin-embedded, sectioned (4 μm), and stained with H&E (Xu et al., 2020). Adipocyte cell size was quantified using the open-source ImageJ software (v1.54), with the AdipoQ plugin (Sie et al., 2022), and lipid-positive areas in Oil Red O–stained liver sections were quantified using ImageJ.

For islet quantification, pancreas was dissected, weighted, fixed, embedded in paraffin, and consecutively cut into 4-μm–thick sections. Sections from the pancreatic tail were stained with H&E, and both islet area and total tissue area were measured using ImageJ software (v1.54).

### 2.13 Oil red O staining

Fresh liver samples were embedded in optimal cutting temperature compound (#4583; Sakura Finetek), snap-frozen at −80 °C, and sectioned at 8 μm using a cryostat (Leica CM 1950). Sections were fixed in 4% paraformaldehyde, stained with Oil Red O working solution (0.5% in isopropanol, diluted 3:2 with water), washed in 60% isopropanol, counterstained with hematoxylin, and imaged (Olympus VS200).

### 2.14 Mito-tracker staining

Cells were stained with 200 nM Mito-Tracker Red CMXRos (C1035; Beyotime) and Hoechst 33342 (C10310-3; RiboBio) per manufacturer’s instructions. Images were acquired with a Zeiss LSM 880 confocal microscope, and signal intensity was quantified in ImageJ (v1.54).

### 2.15 Hepatic triglyceride and total cholesterol measurement

Liver samples (50 mg) were homogenized in ethanol, centrifuged, and supernatants analyzed with commercial kits (Triglyceride, GPO-PAP; Cholesterol, COD-PAP; Nanjing Jiancheng). Absorbance was read at 510 nm (BioTek Synergy H1; Agilent), and values were normalized to protein content.

### 2.16 Transmission electron microscopy (TEM)

Isolated islets were cultured overnight, fixed in 3% glutaraldehyde at 4 °C for >24 h, post-fixed in 1% osmium tetroxide, dehydrated through graded acetone, and embedded in Epon812. Ultrathin sections (∼60 nm) were examined with a JEM-1400FLASH TEM at 6,000× magnification. Image acquisition and mitochondrial morphology analyses were performed in ImageJ (v1.54).

### 2.17 RNA sequencing (RNA-seq) and bioinformatic analysis

RNA-seq was performed by Novogene (Beijing, China) using islets from DNA2^INS2−/−^ and DNA2^fl/fl^ mice after 5 days of HFD feeding. RNA quality was assessed with an Agilent 5400 Fragment Analyzer. Libraries were prepared with the NEBNext® Ultra™ RNA Library Prep Kit and sequenced on an Illumina NovaSeq 6000 (150 bp paired-end). Reads were quality-checked (FastQC), trimmed (Trimmomatic), and aligned to the mouse reference genome (GRCm39) with HISAT2. Gene counts were obtained with featureCounts, differentially expressed genes (DEGs) were identified with DESeq2 (R). Genes with a *p* value ≤0.05 and an absolute fold change ≥1.5 were defined as DEGs, while a more stringent cutoff (|fold change| ≥ 2, *p* ≤ 0.05) was applied for selected downstream analyses. KEGG pathway enrichment was analyzed and visualized with ggplot2.

### 2.18 Statistical analysis

Data are presented as the mean ± SD from at least three independent experiments. Statistical analysis was performed with GraphPad Prism 10. Group comparisons were carried out using unpaired two-tailed Student’s t-test, two-way ANOVA for multi-factorial analyses, or the Kruskal–Wallis test for non-parametric data. Statistical significance was defined as p ≤ 0.05.

## 3 Results

### 3.1 β cell-specific DNA2 deletion does not disturb metabolic homeostasis under physiological conditions

**FIGURE 1:**
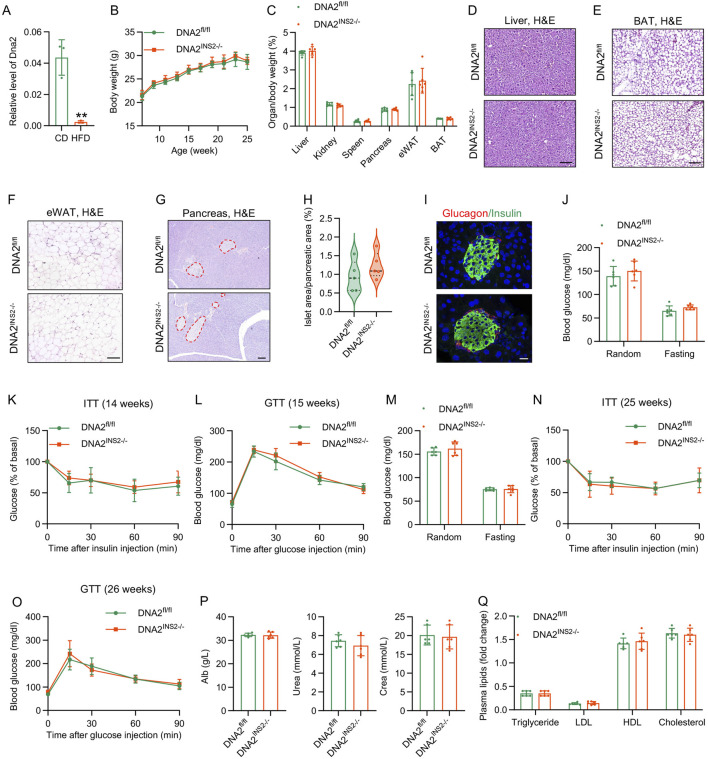
β cell-specific deletion of DNA2 does not affect metabolic phenotype in mice under CD condition. **(A)** Real-time quantitative reverse transcription PCR (RT-qPCR) analysis of DNA2 expression in islets (n = 3 mice per group). **(B)** Body weight gain of DNA2^fl/fl^ and DNA2^INS2−/−^ mice (n = 6 mice per group). **(C)** Organ-to-body weight ratios of major tissues, including liver, spleen, brown adipose tissue (BAT), epididymal white adipose tissue (eWAT), pancreas, and kidney, in 26-week-old mice fed a chow diet (CD) (n = 6 mice per group). **(D–H)** Representative hematoxylin and eosin (H&E)‐stained sections of the liver **(D)**, BAT **(E)**, eWAT **(F)**, and pancreas **(G)** from 26-week-old DNA2^fl/fl^ and DNA2^INS2−/−^ mice, Scale bar, 100 μm. **(H)** Quantification of islet area as the percentage of total pancreatic area (n = 5–6 mice per group). **(I)** Representative immunofluorescence staining (IF) of pancreatic sections from 26-week-old CD-fed mice (n = 3 mice per group). Insulin (green), glucagon (red), and nuclei (DAPI, blue). Scale bar, 20 μm. **(J)** Random and fasting blood glucose levels at 14 weeks of age (n = 6–7 mice per group). **(K)** Insulin tolerance test (ITT) at 14 weeks of age under CD feeding (insulin 1 U/kg body weight, n = 6 mice per group). **(L)** Glucose tolerance test (GTT) at 15 weeks of age under CD feeding (D-glusoce 2 g/kg body weight, n = 6 mice per group). **(M)** Random and fasting blood glucose at 25 weeks of age (n = 6 mice per group). **(N)** ITT at 25 weeks of age (insulin 1 U/kg body weight, n = 6 mice per group). **(O)** GTT at 26 weeks of age (D-glucose 2 g/kg body weight, n = 6 mice per group). **(P,Q)** Plasma biochemical parameters in 26-week-old mice (n = 6 mice per group), including albumin, urea, creatinine, cholesterol, triglycerides, high-density lipoprotein (HDL), low-density lipoprotein (LDL). Data are presented as the mean ± SD; ∗*p* ≤ 0.05, ∗∗*p* ≤ 0.01, and ∗∗∗*p* ≤ 0.001. Significance is assessed using unpaired two-tailed Student’s t-test **(A,C,H,J,M,P,Q)** or two-way ANOVA **(B,K,L,N,O)**.

To assess the physiological role of DNA2 in β cell biology, we first examined its expression in islets under insulin-resistant stress. qPCR revealed a significant downregulation of DNA2 mRNA in pancreatic islets isolated from mice subjected to prolonged HFD feeding than that in islets from controls (Figure 1A), indicating a pathogenic role of DNA2 in β cell dysfunction and diabetes.

To test this idea, we generated a β cell-specific DNA2 knockout mouse model (DNA2^INS2−/−^) using Ins2-Cre-driven recombinase through homologous recombination-mediated genome editing (Supplementary Figure S1A). As expected, DNA2 expression was specifically downregulated in the islets of DNA2^INS2−/−^ mice, with no detectable alterations in other tissues, such as the thymus or hypothalamus (Supplementary Figure S1). DNA2^INS2−/−^ mice were born at Mendelian frequencies and appeared morphologically indistinguishable from their control littermates (DNA2^fl/fl^). Under standard CD conditions, DNA2^INS2−/−^ mice exhibited no significant differences in body weight, organ-to-body weight ratios, or tissue morphology compared with that of DNA2^fl/fl^ control mice (Figures 1B–F). Interestingly, DNA2^INS2−/−^ mice had an increased pancreatic islet area, although the difference was not statistically significant (Figures 1G,H). IF microscopy confirmed that β cell-specific DNA2 deletion did not affect the pancreatic islet architecture (Figure 1I). These results indicate that DNA2 deletion in β cells does not affect normal growth or development.

At 14 and 25 weeks of age, blood glucose levels were not significantly different between the two genotypes under either random or fasting conditions (Figures 1J,M). GTT and ITT analyses showed no significant differences in glucose homeostasis between the groups (Figures 1K,L,N,O). Plasma metabolic profiling confirmed that DNA2 deletion in β cells had no significant effect on circulating metabolic parameters, including albumin, urea, creatinine, triglycerides, low-density lipoprotein (LDL), high-density lipoprotein (HDL), and total cholesterol (T-cholesterol) levels (Figures 1P,Q). These data, together with the increased glucose-stimulated insulin secretion observed *in vivo*, suggest that β cell DNA2 deletion may enhance acute insulin secretion without perturbing systemic metabolic homeostasis under physiological conditions.

### 3.2 β cell-specific deletion of DNA2 exacerbates HFD-induced metabolic dysregulation

**FIGURE 2:**
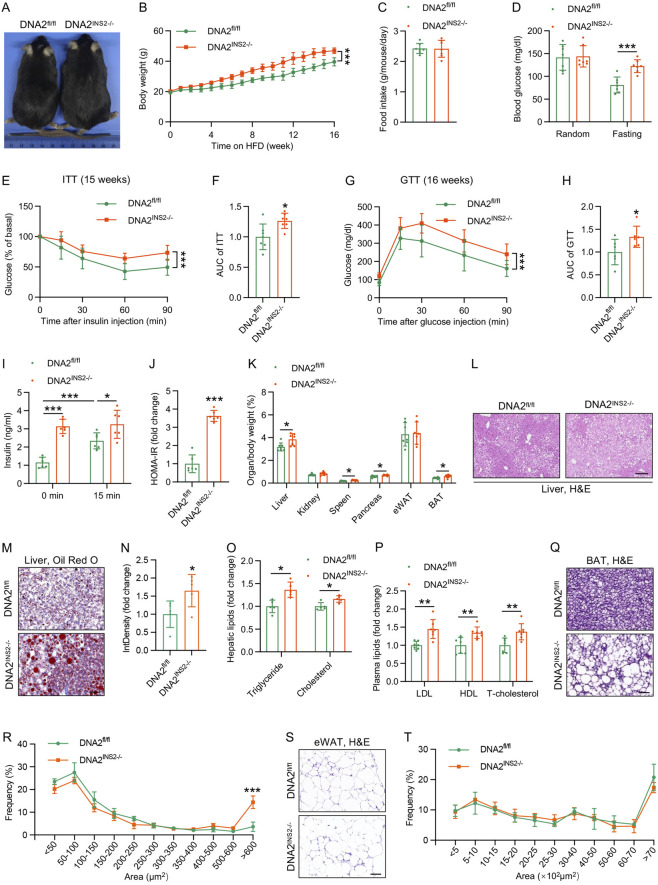
β cell-specific deletion of DNA2 exacerbates HFD-induced metabolic dysfunction. **(A)** Representative images of DNA2^INS2−/−^ and DNA2^fl/fl^ after 16 weeks of HFD feeding. **(B)** Body weight gain during HFD feeding (n = 7 mice per group). **(C)** Average daily food intake during HFD feeding (n = 7 mice per group). **(D)** Random and fasting blood glucose levels after 15 weeks of HFD feeding (n = 7 mice per group). **(E,F)** ITT and AUC after 15 weeks of HFD feeding (insulin 1 U/kg body weight, n = 7 mice per group). **(G,H)** GTT and AUC after 16 weeks of HFD feeding (D-glucose 1 g/kg body weight, n = 6 mice per group). **(I)** Plasma insulin levels measured during GTT (n = 6 mice per group). **(J)** HOMA-IR in obese mice (n = 6 mice per group). **(K)** Organ-to-body weight ratios of major tissues in mice after 16 weeks of HFD feeding (n = 7 mice per group), including liver, spleen, BAT, eWAT, pancreas, and kidney. **(L)** Representative images of H&E-stained liver paraffin sections after 16 weeks of HFD feeding (n = 4 mice per group). Scale bar, 50 μm. **(M,N)** Representative Oil Red O–stained liver cryosections from mice after 16 weeks of HFD feeding (n = 4 mice per group) and quantification of lipid-positive area. Scale bar, 50 μm. **(O)** Quantification of hepatic triglyceride and cholesterol levels (n = 5 mice per group). **(P)** Plasma lipid profiles after 16 weeks of HFD feeding (n = 7 mice per group), including LDL, HDL, and total cholesterol. **(Q,R)** Representative H&E‐stained sections of the BAT from mice after 16 weeks of HFD feeding and quantification of adipocyte size (n = 4 mice per group). Scale bar, 50 μm. **(S,T)** Representative H&E‐stained sections of the eWAT from mice after 16 weeks of HFD feeding and quantification of adipocyte size (n = 4 mice per group). Scale bar, 50 μm. Data are presented as the mean ± SD; ∗*p* ≤ 0.05, ∗∗*p* ≤ 0.01, and ∗∗∗*p* ≤ 0.001. Significance is assessed using unpaired two-tailed Student’s t-test **(C,D,F,H,I–K,N–P)** or two-way ANOVA **(B,E,G,R,T)**.

We assessed the effects of β cell-specific DNA2 deletion on HFD-induced metabolic disturbances. Before HFD feeding, DNA2^INS2−/−^ mice exhibited comparable body weight, glycemic levels, and glucose tolerance to those of their littermate controls (Supplementary Figure S2). Upon HFD challenge, DNA2^INS2−/−^ mice gained significantly more body weight than that of DNA2^fl/fl^ mice, despite similar food intake (Figures 2A–C), indicating an increased susceptibility to diet-induced obesity. Additionally, these mice demonstrated increased fasting blood glucose levels at both 8 and 16 weeks of HFD feeding (Figure 2D; Supplementary Figure S3A). Moreover, DNA2^INS2−/−^ mice exhibited impaired glucose tolerance and insulin sensitivity, as assessed by GTT and ITT, respectively (Figures 2E–H; Supplementary Figures S3B–E). Notably, the plasma insulin levels were significantly increased in DNA2^INS2−/−^ mice both under fasting and glucose-inducing conditions (Figure 2I; Supplementary Figure S3F), accompanied by significantly higher homeostasis model assessment of insulin resistance (HOMA-IR) indices (Figure 2J; Supplementary Figure S3G). These findings indicate that β cell-specific deletion of DNA2 exacerbates glucose intolerance, hyperinsulinemia, insulin resistance, and overall metabolic deterioration in HFD-induced obesity.

### 3.3 β cell DNA2 deletion exacerbates ectopic lipid deposition in HFD-induced obesity

Because DNA2^INS2−/−^ mice exhibit exacerbated obesity and insulin resistance, we assessed whether lipid metabolism and distribution were similarly perturbed. Organ analysis demonstrated that HFD-fed DNA2^INS2−/−^ mice had significantly increased liver, spleen, pancreas, and brown adipose tissue (BAT) weights compared to that of the controls (Figure 2K). H&E and Oil Red O staining revealed significant lipid droplet accumulation in the livers of DNA2^INS2−/−^ mice, indicating severe hepatic steatosis (Figures 2L–N). Quantitative lipid analysis confirmed increased hepatic triglyceride and cholesterol content in DNA2^INS2−/−^ mice (Figure 2O). Consistently, plasma lipid profiling revealed increased levels of LDL, HDL, and T-cholesterol in DNA2^INS2−/−^ mice (Figure 2P), indicating systemic dysregulation of lipid metabolism. Furthermore, BAT from DNA2^INS2−/−^ mice displayed marked whitening, evidenced by excessive lipid deposition and altered cellular morphology relative to DNA2^fl/fl^ controls, whereas eWAT remained largely comparable between the two groups (Figures 2Q–T). These findings demonstrate that β cell-specific DNA2 deletion induces ectopic lipid deposition and impairs systemic lipid homeostasis under HFD conditions.

### 3.4 DNA2 deletion facilitates β cell proliferation

**FIGURE 3:**
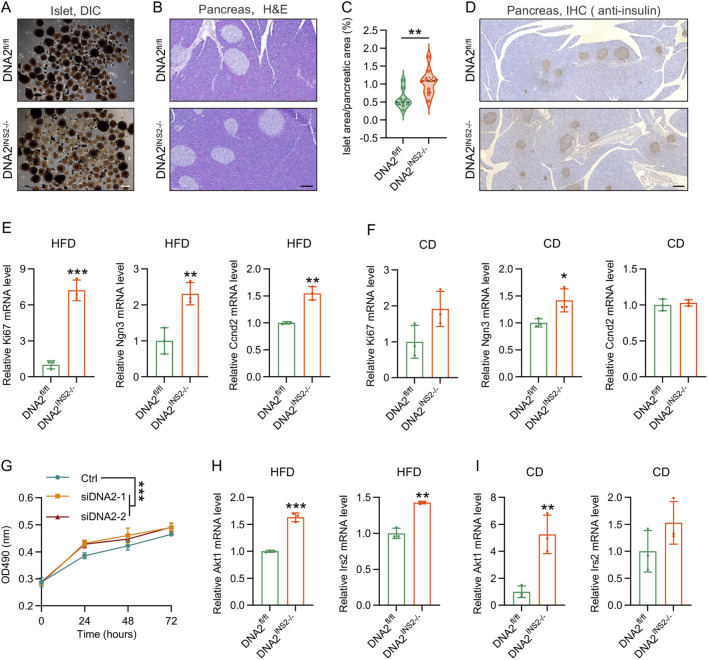
Deletion of DNA2 promotes β cell proliferation. **(A)** Representative images of isolated mouse islets (n = 3 mice per group). Scale bar, 200 μm. **(B)** Representative H&E-stained pancreatic sections from mice after 16 weeks of HFD feeding (n = 4 mice per group). Scale bar, 100 μm. **(C)** Quantification of islet area illustrated in **(B)** as a percentage of total pancreatic area (n = 8–9 sections per group). **(D)** Representative insulin immunohistochemistry (IHC) staining of pancreatic sections from mice after 16 weeks of HFD feeding (n = 4 mice per group). Scale bar, 200 μm. **(E,F)** mRNA expression of proliferation-related genes in islets from HFD-(16 weeks, **(E)**) and CD- (16 weeks, **(F)**) fed mice (n = 3 mice per group). **(G)** Cell viability in Min6 cells transfected with control or DNA2 small interfering RNA (siRNA) measured by MTS assay (n = 5 replicates per group). **(H,I)** mRNA levels of hypertrophy-related genes in islets from HFD-(16 weeks, **(H)**) and CD-(16 weeks, **(I)**) fed mice (n = 3 mice per group). Data are presented as the mean ± SD; ∗*p* ≤ 0.05, ∗∗*p* ≤ 0.01, and ∗∗∗*p* ≤ 0.001. Significance is assessed using unpaired two-tailed Student’s t-test **(C,E,F,H,I)** or two-way ANOVA **(G)**.

Circulating insulin levels are controlled by a combination of the number of β cells and the insulin secretory capacity of individual β cells. We first determined the effect of DNA2 deletion on β cell mass. We next examined β cell mass and proliferation to investigate whether these functional changes were accompanied by alterations in islet morphology. DNA2^INS2−/−^ mice consistently exhibited a greater number and larger size of islets compared to that of their littermate controls (Figure 3A). H&E staining and insulin IHC revealed that HFD-induced islet hyperplasia was significantly more pronounced in DNA2^INS2−/−^ mice (Figures 3B–D). Obesity-induced hyperplasia is commonly associated with increased β cell proliferation (Hu et al., 2022; Kubo et al., 2025; Chen et al., 2025). Therefore, we assessed the expression of hypertrophy- and proliferation-related genes in the isolated islets. Notably, DNA2^INS2−/−^ islets exhibited significantly increased expression of proliferation markers (Kiel 67 antigen [Ki67], cyclin D2 [Ccnd2], and neurogenin 3 [Ngn3]) following HFD feeding, with only modest increases observed under CD conditions (Figures 3E,F). Consistent with this *in vivo* phenotype, DNA2 knockdown significantly enhanced the proliferation of Min6 β cells (Figure 3G). Similarly, HFD-fed DNA2^INS2−/−^ islets significantly upregulated hypertrophy-associated genes (Akt1 and Irs2), whereas under CD conditions, only an increase in Akt1 expression was observed (Figures 3H,I).

These findings indicate that DNA2 acts as a negative regulator of β cell proliferation, and its deficiency facilitates obesity-associated islet expansion, potentially serving as a compensatory response to meet the increased metabolic demand under nutritional stress.

### 3.5 DNA2 deletion enhances insulin secretion without affecting insulin production

**FIGURE 4:**
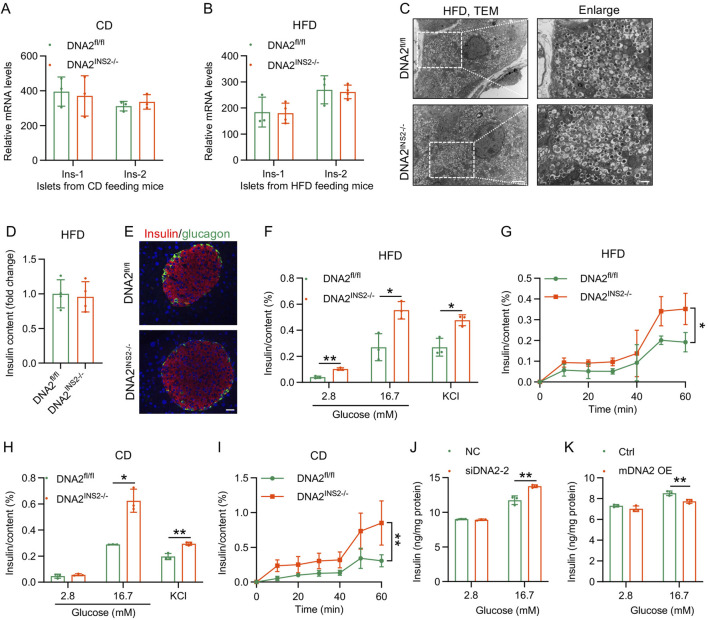
β cell-specific deletion of DNA2 promotes insulin secretion without impairing insulin production. **(A,B)** Relative mRNA expression of Ins1 and Ins2 in isolated islets from mice fed with CD (16 weeks, **(A)**) and HFD (16 weeks, **(B)**) (n = 3–4 mice per group). **(C)** Representative transmission electron microscopy (TEM) images of β cells from mice after 16 weeks of HFD feeding (n = 3 mice per group). Scale bar: 2 μm (left) and 500 nm (right). **(D)** Total insulin content in islets isolated from mice after 16 weeks of HFD feeding measured by ELISA (n = 4 mice per group). **(E)** Representative IF of pancreatic sections from mice after 16 weeks of HFD feeding (n = 4 mice per group). Insulin (red), glucagon (green), and nuclei (DAPI, blue). Scale bar, 20 μm. **(F)** Glucose- and KCl-stimulated insulin secretion from islets isolated from HFD-fed mice for 16 weeks (n = 3 replicates per group). **(G)** Time-course analysis of glucose-stimulated insulin secretion (GSIS) in islets isolated from HFD-fed mice for 16 weeks. Islets are stimulated with 16.7 mM glucose, and insulin secretion is measured at the indicated time points (n = 3–4 replicates per group). **(H)** Glucose- and KCl-stimulated insulin secretion in islets isolated from mice fed a CD for 16 weeks (n = 3 replicates per group). **(I)** Time-course analysis of GSIS in islets in islets isolated from mice fed a CD for 16 weeks (n = 3 replicates per group). **(J)** GSIS assay in INS-1 cells transfected with control or DNA2 siRNA for 48 h (n = 3 replicates per group). **(K)** GSIS assay in INS-1 cells transfected with empty vector or mDNA2 overexpression plasmid for 48 h (n = 3 replicates per group). Data are presented as the mean ± SD; ∗*p* ≤ 0.05, ∗∗*p* ≤ 0.01, and ∗∗∗*p* ≤ 0.001. Significance is assessed using unpaired two-tailed Student’s t-test **(A,B,D,F,H,J,K)** or two-way ANOVA **(G,I)**.

We next evaluated the consequences of DNA2 deficiency on β cell functionality, specifically insulin biosynthesis and secretion. Remarkably, despite the presence of hyperglycemia and elevated circulating insulin in DNA2^INS2−/−^ mice (Figure 2I; Supplementary Figure S3F), there was no significant alteration in the islet mRNA expression of insulin (Ins-1 and Ins-2) under either CD or HFD conditions (Figures 4A,B). Consistently, ultrastructural analysis demonstrated comparable morphology and abundance of insulin granules between the genotypes (Figure 4C), and the total insulin content per islet mass was unaffected (Figure 4D). Moreover, IF imaging confirmed the preserved islet architecture and α/β cell organization (Figure 4E), indicating that insulin biosynthesis and storage were intact.

Subsequently, we assessed the *ex vivo* insulin secretory capacity of the isolated islets. Following chronic HFD exposure, both glucose- and KCl-stimulated insulin release were significantly augmented in DNA2-deficient islets compared with that in controls (Figure 4F). Dynamic perfusion analysis revealed that the first and second phases of insulin secretion were amplified in DNA2-depleted islets (Figure 4G). These observations were recapitulated under physiological dietary conditions (Figures 4H,I). Extending these findings to the INS-1 β cell line, siRNA-mediated DNA2 knockdown significantly potentiated insulin secretion, whereas transient overexpression attenuated it (Figures 4J,K). In summary, β cell DNA2 depletion consistently hypersensitizes stimulus-secretion coupling across experimental models without altering insulin biosynthesis and content.

### 3.6 DNA2 limits β cell mitochondrial activity and quality

**FIGURE 5:**
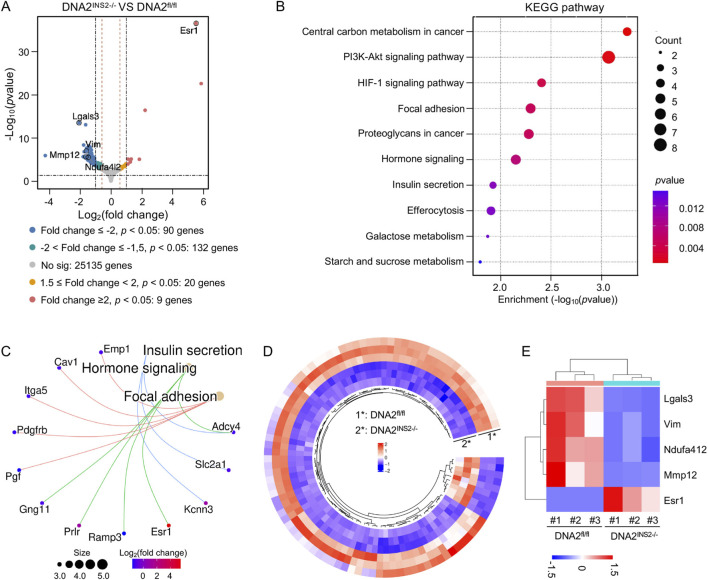
Transcriptomic alterations in DNA2-deficient islets. **(A)** Volcano plot demonstrating differentially expressed genes (DEGs) in islets from DNA2^INS2−/−^ mice compared to that in DNA2^fl/fl^ littermate controls after 5 days of high-fat diet (HFD) feeding (n = 3 mice per group). Among the upregulated genes, nine exhibited a fold change ≥2 and 20 exhibited a fold change between 1.5 and 2. Among the downregulated genes, 90 exhibited a fold change ≥2 and 132 exhibited a fold change between 1.5 and 2 (*p* ≤ 0.05). Primary genes associated with mitochondrial regulation, including lectin, galactoside-binding, soluble 3 (Lgals3), estrogen receptor 1 (Esr1), NADH: ubiquinone oxidoreductase subunit A4-like 2 (Ndufa4l2), vimentin (Vim), and matrix metallopeptidase 12 (Mmp12), are highlighted. **(B,C)** Kyoto Encyclopedia of Genes and Genomes (KEGG) pathway analysis of 99 DEGs identified by RNA sequencing (|fold change| ≥ 2, *p* ≤ 0.05), visualized by a bubble plot **(B)** highlighting enriched pathways and a cnetplot **(C)** displaying specific genes associated with these terms. **(D)** Circular heatmap demonstrating global gene expression alterations between DNA2^fl/fl^ and DNA2^INS2−/−^ mice islets. Genes are hierarchically clustered based on Z-score-normalized expression values. Red and blue indicate relative upregulation and downregulation, respectively. **(E)** Heatmap highlighting expression of representative DEGs associated with mitochondrial function (Lgals3, Vim, Ndufa4l2, Mmp12, and Esr1) in individual samples. Scale represents log_2_(fold change).

To assess the transcriptional basis underlying the secretory phenotype and capture early molecular responses associated with DNA2 deficiency, we performed transcriptomic profiling to explore the alternative pathways. RNA-seq of islets harvested after 5 days of HFD exposure—a stage that precedes detectable β cell loss—identified 251 DEGs (|fold change| ≥ 1.5, *p* ≤ 0.05) in DNA2^INS2−/−^ islets compared to those in DNA2^fl/fl^ controls. Of these, 99 met a more stringent threshold (|fold change| ≥ 2, *p* ≤ 0.05) (Figures 5A,D). Subsequent KEGG enrichment analysis of these 99 DEGs revealed that DNA2 deletion was associated with stimulus-secretion-coupling pathways, including insulin secretion, hormonal signaling, and focal adhesion (Figures 5B,C), indicating a crucial role in exocytosis. Notably, five DEGs—lectin, galactoside-binding, soluble 3 (Lgals3), estrogen receptor 1 (Esr1), NADH: ubiquinone oxidoreductase subunit A4-like 2 (Ndufa4l2), vimentin, and matrix metallopeptidase 12 (Mmp12)—were implicated in mitochondrial regulation (Li et al., 2022; Huynh et al., 2024; Kubala et al., 2023; Coppin et al., 2020) (Figure 5E), facilitating further assessment of mitochondrial function in DNA2-deficient β cells.

**FIGURE 6:**
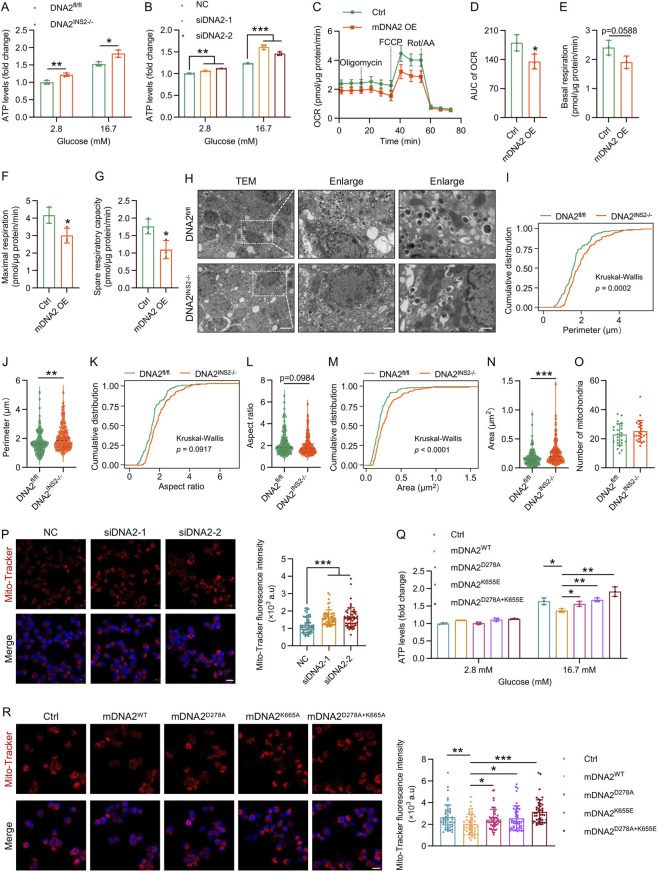
DNA2 suppresses mitochondrial activity in a D278- and K655-dependent manner in pancreatic β cells. **(A)** ATP levels in isolated mouse islets stimulated with glucose (n = 3 replicates per group). **(B)** Glucose-stimulated ATP production in INS-1 cells transfected with control siRNA or DNA2 siRNA for 48 h (n = 3 replicates per group). **(C)** Mitochondrial stress test in INS-1 cells overexpressing mDNA2 (n = 3 replicates per group), using sequential injections of oligomycin (1.5 μM), FCCP (2.5 μM), and rotenone/antimycin A (0.5 μM). **(D–G)** Quantification of Seahorse metabolic parameters in INS-1 cells (n = 3 replicates per group), including area under the curve of oxygen consumption rate **(D)**, basal respiration **(E)**, maximal respiration **(F)**, and spare respiratory capacity **(G)**. **(H–O)** Transmission electron microscopy (TEM) analysis of mitochondrial morphology in β cells from DNA2^fl/fl^ and DNA2^INS2−/−^ mice after 16 weeks of HFD feeding (n = 4 mice per group). **(H)** Representative TEM images. Scale bar: 5 μm (left), 1 μm (middle), and 500 nm (right). **(I–O)** Mitochondrial morphological parameters are demonstrated as cumulative frequency distribution and bar graphs of mitochondrial perimeter **(I,J)**, aspect ratio **(K,L)**, mitochondrial area **(M,N)**, and number of mitochondria **(O)** per cell. **(P)** Representative Mito-Tracker images of INS-1 cells following DNA2 knockdown. Quantification of fluorescence intensity is demonstrated on the right. Scale bar, 10 μm. **(Q)** Glucose-stimulated ATP production in INS-1 cells transfected with mDNA2^WT^, mDNA2^D278A^, mDNA2^K655E^, mDNA2^D278A+K655E^, and empty plasmid. **(R)** Representative images of Mito-Tracker in INS-1 cells transfected with mDNA2^WT^, mDNA2^D278A^, mDNA2^K655E^, mDNA2^D278A+K655E^, and empty plasmid. Quantification of fluorescence intensity is demonstrated on the right. Scale bar, 10 μm. Data are presented as the mean ± SD; ∗p ≤ 0.05, ∗∗p ≤ 0.01, and ∗∗∗p ≤ 0.001. Significance is assessed using unpaired two-tailed Student’s t-test **(A,B,D–G,J,L,N,O–R)** or Kruskal–Wallis test **(I,K,M)**.

Because mitochondrial ATP production is crucial for stimulus-secretion coupling, we assessed mitochondrial performance in both islets and INS-1 cells to determine whether DNA2 affects β cell bioenergetics. Glucose-stimulated ATP production was significantly increased in DNA2-deficient islets and INS-1 cells (Figures 6A,B), whereas DNA2 overexpression reduced ATP production (Figure 6Q). Mitochondrial stress tests using Seahorse XF analysis revealed that DNA2-overexpressing INS-1 cells exhibited reduced basal and maximal respiration, along with reduced spare respiratory capacity (Figures 6C–G), indicating compromised oxidative phosphorylation. TEM of pancreatic β cells from DNA2^INS2−/−^ mice revealed significant mitochondrial hypertrophy, reflected by increased mitochondrial perimeter, aspect ratio, and cross-sectional area (Figures 6H–N), although the mitochondrial density remained unaltered (Figure 6O). Similarly, Mito-Tracker fluorescence intensity was increased in DNA2-deficient INS-1 cells and reduced upon DNA2 overexpression (Figures 6P,R), confirming that DNA2 loss enhanced mitochondrial mass and activity.

To elucidate the enzymatic basis of this regulation, we focused on two catalytic sites of DNA2: the nuclease site D278 and helicase site K655 (Levikova et al., 2013; Pinto et al., 2016; Shen et al., 2022). We generated single-point mutants (mDNA2^D278A^ and mDNA2^K655A^) and a double mutant (mDNA2^D278A+K655A^) and expressed them in INS-1 cells to assess the enzymatic activity of each. Both single mutants partially alleviated the inhibitory effects of wild-type DNA2 (mDNA2^WT^) overexpression on glucose-stimulated ATP production and mitochondrial activity, whereas the double mutant exerted a more significant rescue effect across all parameters (Figures 6Q,R), highlighting the cooperative requirement for both catalytic domains.

In summary, our findings position DNA2 as a molecular gatekeeper of β cell mitochondrial production. Through its coordinated nuclease and helicase activities, DNA2 limits mitochondrial size and activity, thereby restraining ATP production and insulin secretion in response to glucose. In contrast, DNA2 deficiency results in mitochondrial hypertrophy, hyperactivation, and insulin hypersecretion. These data reveal a DNA2-mitochondria-insulin axis that integrates genome maintenance with metabolic plasticity in β cells under nutritional stress.

## 4 Discussion

This study identified DNA2 as a previously unrecognized β cell-intrinsic rheostat of systemic glucose homeostasis. Although DNA2 is known for its role in mtDNA replication and repair (Zheng et al., 2020; Zheng et al., 2008), our integrative *in vivo* and *in vitro* analyses indicate that it negatively regulates both β cell proliferation and insulin secretion by limiting mitochondrial bioenergetics. Under normal dietary conditions, β cell-specific DNA2 deletion did not affect body weight, glucose tolerance, or insulin sensitivity but significantly increased circulating insulin levels in response to glucose stimulation. However, the metabolic consequences of DNA2 deficiency were exacerbated under metabolic stress. Following HFD feeding, DNA2^INS2−/−^ mice exhibited greater weight gain, glucose intolerance, insulin resistance, ectopic lipid deposition, and significant hyperinsulinemia. These phenotypes align with emerging evidence that chronic compensatory hyperinsulinemia is not merely a benign adaptation but a primary pathophysiological association between insulin resistance and overt T2D with its cardiometabolic sequelae (van Vliet et al., 2020; Houston and Templeman, 2025; Janssen, 2021). Our findings revealed that the loss of β cell DNA2 exacerbates systemic hyperinsulinemia and downstream metabolic dysfunction through two synergistic processes: (i) enhanced β cell proliferation and islet hypertrophy and (ii) augmented glucose-stimulated insulin secretion independent of increased proinsulin biosynthesis.

Mechanistically, we identified that DNA2 restrains mitochondrial function in β cells. DNA2-deficient β cells exhibited increased mitochondrial respiration and ATP production, accompanied by mitochondrial enlargement and increased Mito-Tracker staining intensity, indicating an enhanced mitochondrial activity. In contrast, DNA2 overexpression in INS-1 cells suppressed oxidative phosphorylation, and domain mutation analysis revealed that both the nuclease and helicase activities of DNA2 contribute to this regulatory effect. Together, these findings establish that DNA2 exerts a robust regulatory effect on mitochondrial oxidative phosphorylation at the functional level, as reflected by coordinated changes in oxygen consumption, ATP production, mtDNA content, and mitochondrial morphology. Recent studies have suggested that DNA2 may influence the activity of specific mitochondrial respiratory complexes, including complex II (Liu et al., 2024). In the present study, we focused on global mitochondrial oxidative phosphorylation rather than resolving individual respiratory complex activities or substrate-specific pathways. Future studies employing complex-specific respiratory assays and complementary biochemical approaches will be important to determine whether DNA2 preferentially regulates distinct OXPHOS complexes in β cells and other metabolic tissues. In addition, although mitochondrial ultrastructural alterations were consistently observed in DNA2-deficient β cells, future studies focusing specifically on mitochondrial morphology may benefit from optimized fixation strategies to further improve ultrastructural preservation and image resolution (Faitg et al., 2020; Qin et al., 2021), particularly in analyses aimed at resolving fine mitochondrial architecture.

Although DNA2 is canonically defined as a key factor in mtDNA replication and repair, our findings revealed a distinct role in pancreatic β cells. Instead of causing evident mitochondrial genomic instability, β cell–specific DNA2 deletion primarily enhanced mitochondrial respiration and insulin secretion. These observations suggest that β cells may harbor compensatory genome-maintenance mechanisms that preserve mitochondrial stability in the absence of DNA2. Candidate factors reported in other cell types include mitochondrial genome maintenance exonuclease 1 (MGME1), PIF1, suv3 like RNA helicase (SUPV3L1), and RecQ-like helicase 4 (RECQ4) (Kornblum et al., 2013; Pawlowska et al., 2017), although their activity in β cells under metabolic stress remains to be determined. Collectively, these results highlight a noncanonical function of DNA2 as a negative regulator of β cell bioenergetics and secretory activity, illustrating how its role in β cells differs from other cell types and revealing a potential molecular brake on nutrient-driven β cell overactivation.

Notably, the number of DEGs identified by RNA-seq was relatively limited, even under a moderate cutoff threshold. This pattern indicates that under HFD conditions, DNA2 may affect β cell function primarily by modulating cellular energetics—specifically mitochondrial activity—rather than inducing broad transcriptional reprogramming. Although no overt enrichment of mitochondrial pathways was detected, several DEGs (Lgals3, Esr1, and Ndufa4l2) have documented roles in mitochondrial regulation, indicating a more selective transcriptional effect. Importantly, the present transcriptomic analysis was performed at an early stage of HFD challenge (5 days), prior to overt β-cell failure or systemic metabolic deterioration. It therefore remains possible that prolonged metabolic stress during chronic HFD feeding could elicit more extensive transcriptional remodeling, including sustained alterations in insulin signaling and metabolic pathways in both β cells and peripheral tissues such as the liver. Future studies incorporating RNA-seq analyses under chronic diet-induced obesity conditions will be instrumental in defining the temporal evolution of DNA2-dependent transcriptional programs and their contribution to long-term metabolic dysfunction. Moreover, the mechanisms associating DNA2 with transcriptional regulation in β cells remain unclear and require future assessment, potentially involving metabolic intermediates, signaling pathways, and mitochondrial-nuclear communication.

The association between mitochondrial overactivation and β cell hyperfunction has been increasingly recognized (Chareyron et al., 2020; Wu et al., 2017). Mitochondria-derived ATP is the primary driver of insulin secretion, and excessive mitochondrial activity can potentiate insulin release, even in the absence of alterations in glucose metabolism (Jezek et al., 2022). Our findings align with this concept and provide direct evidence that the modulation of mitochondrial dynamics by DNA2 affects β cell production. Additionally, because mitochondrial signals modulate cell growth, the enhanced β cell proliferation in DNA2-deficient mice is likely, at least in part, mitochondrially driven. Supporting this, we observed increased islet size and number, along with the upregulation of genes involved in β cell growth, such as Akt1, Irs2, and Ki67. Notably, although Akt1 expression was modestly increased in CD-fed DNA2^INS2−/−^ islets, whether this alteration has long-term biological or pathological relevance under physiological conditions remains unclear. In addition, while β-cell proliferation contributes to increased β-cell mass under metabolic stress, other mechanisms, such as β-cell dedifferentiation, may also play an important role (Son and Accili, 2023). Classical markers of β-cell dedifferentiation were not directly assessed in the present study, so its contribution cannot be formally excluded. Future studies are warranted to directly investigate the role of dedifferentiation in islet remodeling.

However, crucial mechanistic questions remain. For instance, our transcriptomic and functional analyses identified a set of downstream genes whose expression is altered upon β cell–specific loss of DNA2, linking DNA2 to the control of mitochondrial function and bioenergetics. Rather than proposing additional candidate effectors, an important next step is to define the molecular cascade that connects DNA2 to the regulation of these validated targets. Our results demonstrate that DNA2 regulates β-cell function by modulating the expression of nuclear-encoded mitochondrial genes. This suggests that DNA2 may act at the nuclear level to influence mitochondrial activity, rather than directly targeting the mitochondrial genome. One plausible mechanism is that DNA2, through its canonical role in DNA replication and repair, may alter chromatin accessibility or transcriptional fidelity of genes required for mitochondrial metabolism (Zheng et al., 2020). Alternatively, DNA2 deficiency might trigger nuclear stress responses that reprogram mitochondrial function (Ryan and Hoogenraad, 2007), thereby aligning cellular energy production with the heightened insulin secretory demand of β cells. In addition, the Ins2-Cre driver has been reported to exhibit low-level activity in certain hypothalamic neurons and potentially influence appetite regulation (Wicksteed et al., 2010). In our study, comparable food intake (Figure 2C) and DNA2 expression in hypothalamus (Supplementary Figure S1E) between DNA2^fl/fl^ and DNA2^INS2−/−^ mice, together with our previous studies using the Ins2-Cre driver (Xu et al., 2020), argued against a neuronal contribution. Nevertheless, a minor effect of neuronal DNA2 deletion cannot be completely excluded, which warrants further investigation.

In conclusion, this study reveals a previously unrecognized role of DNA2 as a key β cell regulator that limits mitochondrial bioenergetics and insulin secretion. The loss of this restraint initiates a feed-forward cycle of β cell proliferation and hypersecretion, shifting physiological compensation to pathological hyperinsulinemia under metabolic stress. Future studies should explore the upstream nutrient-sensing pathways and compensatory mitochondrial quality control networks that compensate for DNA2 deficiency. These insights may support novel therapeutic strategies to restore β cell metabolic balance and delay or prevent T2D onset.

## Data Availability

The raw sequence data reported in this paper have been deposited in the Genome Sequence Archive (Genomics, Proteomics & Bioinformatics 2025) in National Genomics Data Center (Nucleic Acids Res 2025), China National Center for Bioinformation / Beijing Institute of Genomics, Chinese Academy of Sciences (GSA: CRA039040) that are publicly accessible at https://ngdc.cncb.ac.cn/gsa.

## Supplementary material

SUPPLEMENTARY FIGURE S1 Generation of β cell-specific DNA2 knockout mice. **(A)** Schematic illustration of the generation of β cell-specific DNA2 knockout mice. **(B)** Genotyping of islets demonstrating a 210 bp band corresponding to β cell-specific deletion of DNA2. **(C–E)** mRNA levels of DNA2 in the islets **(C)**, thymus **(D)**, and hypothalamus **(E)** (n = 3 mice per group). Data are presented as the mean ± SD; **p* ≤ 0.05, ***p* ≤ 0.01, and ****p* ≤ 0.001. Significance is assessed using unpaired two-tailed Student’s t-test **(C–E)**.

SUPPLEMENTARY FIGURE S2 Baseline metabolic parameters in DNA2^INS2−/−^ versus control mice prior to HFD intervention. **(A–C)** Before HFD feeding, 6-week-old mice were assessed for body weight **(A)** (n = 10), random and fasting blood glucose levels **(B)** (n = 7 mice per group), and glucose tolerance test **(C)** (2 g/kg body weight, n = 7 mice per group). Data are presented as the mean ± SD; **p* ≤ 0.05, ***p* ≤ 0.01, and ****p* ≤ 0.001. Significance is assessed using unpaired two-tailed Student’s t-test **(A, B)** or two-way ANOVA **(C)**.

SUPPLEMENTARY FIGURE S3 β cell-specific deletion of DNA2 exacerbates HFD-induced metabolic dysfunction. **(A)** Random and fasting blood glucose levels after 8 weeks of HFD feeding (n = 7 mice per group). **(B,C)** ITT and AUC after 8 weeks of HFD feeding (insulin 1 U/kg body weight, n = 7 mice per group). **(D,E)** GTT and AUC after 9 weeks of HFD feeding (D-glucose 1 g/kg body weight, n = 6 mice per group). **(F)** Plasma insulin levels measured during GTT (n = 6 mice per group). **(G)** HOMA-IR after 8 weeks of HFD feeding (n = 6 mice per group). Data are presented as the mean ± SD; **p* ≤ 0.05, ***p* ≤ 0.01, and ****p* ≤ 0.001. Significance is assessed using unpaired two-tailed Student’s t-test **(A,C,E–G)** or two-way ANOVA **(B,D)**.
