# Supplementary figures and images for: DNA2 acts as a brake on β cell insulin hypersecretion and diet-induced metabolic dysfunction
Source: Front Cell Dev Biol. 2026 Mar 4;14:1733190. doi: 10.3389/fcell.2026.1733190 (PMC12996210; doi:10.3389/fcell.2026.1733190)

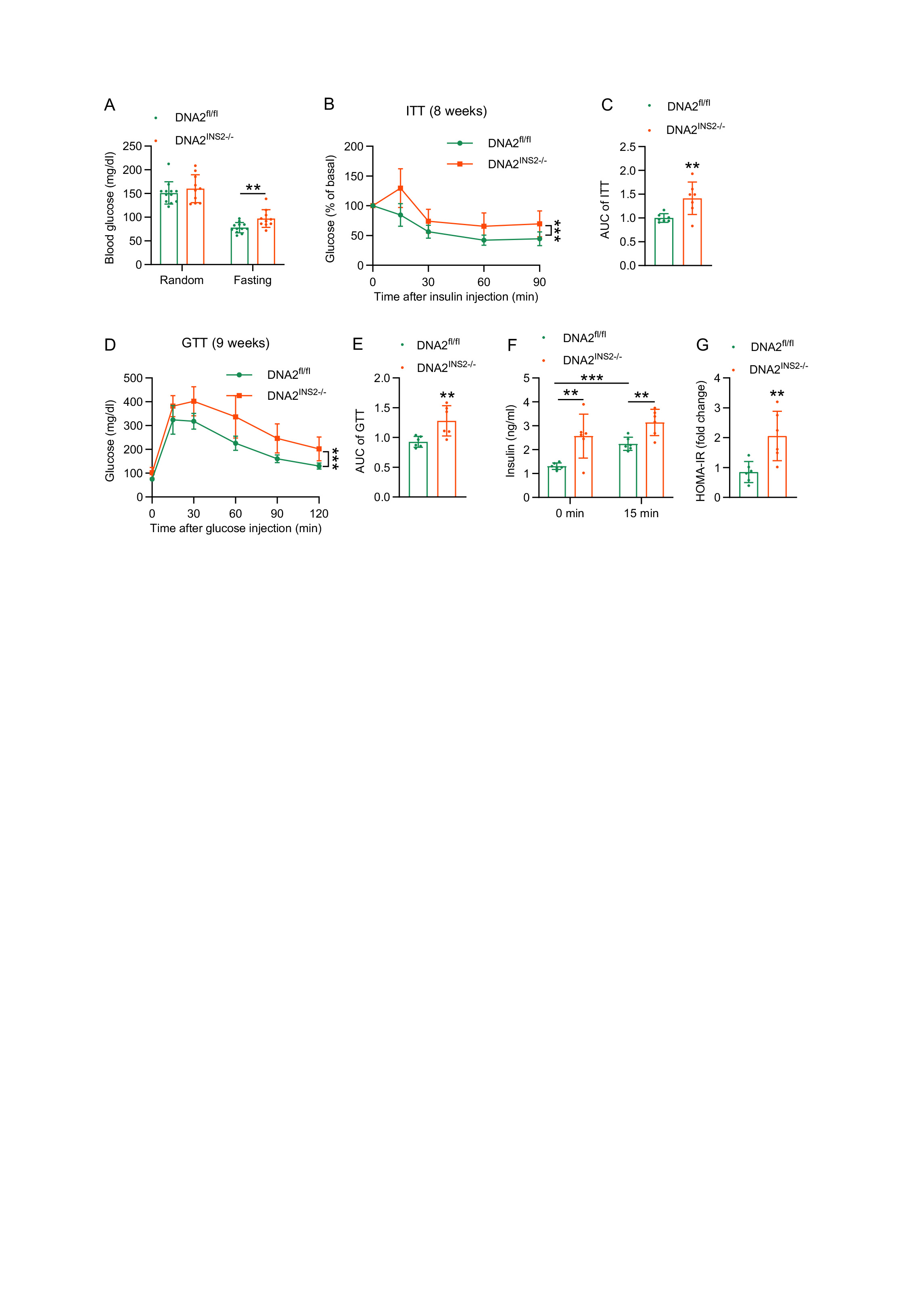

Supplement: Supplementary file 1 [file Image3.jpeg]

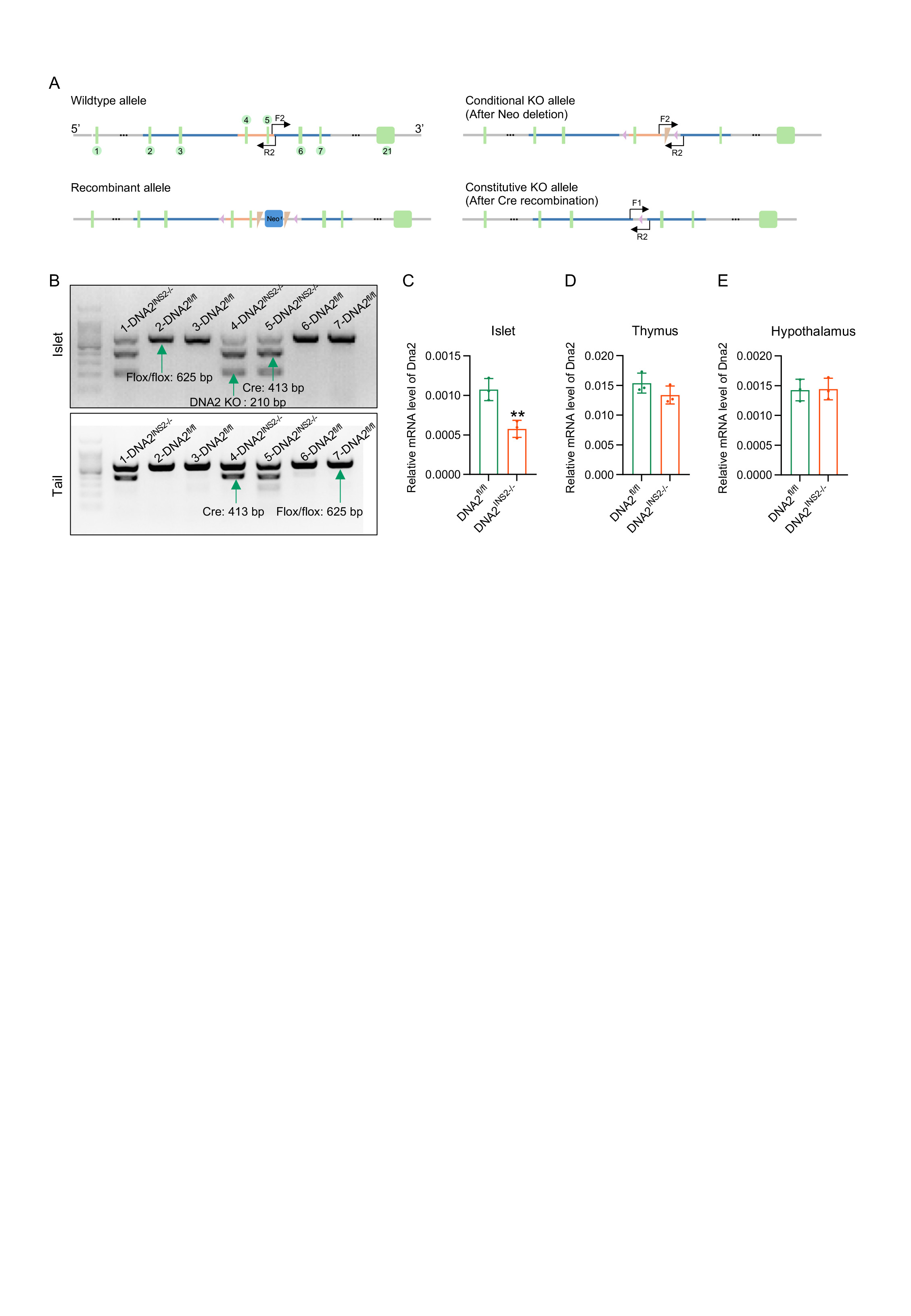

Supplement: Supplementary file 3 [file Image1.jpeg]

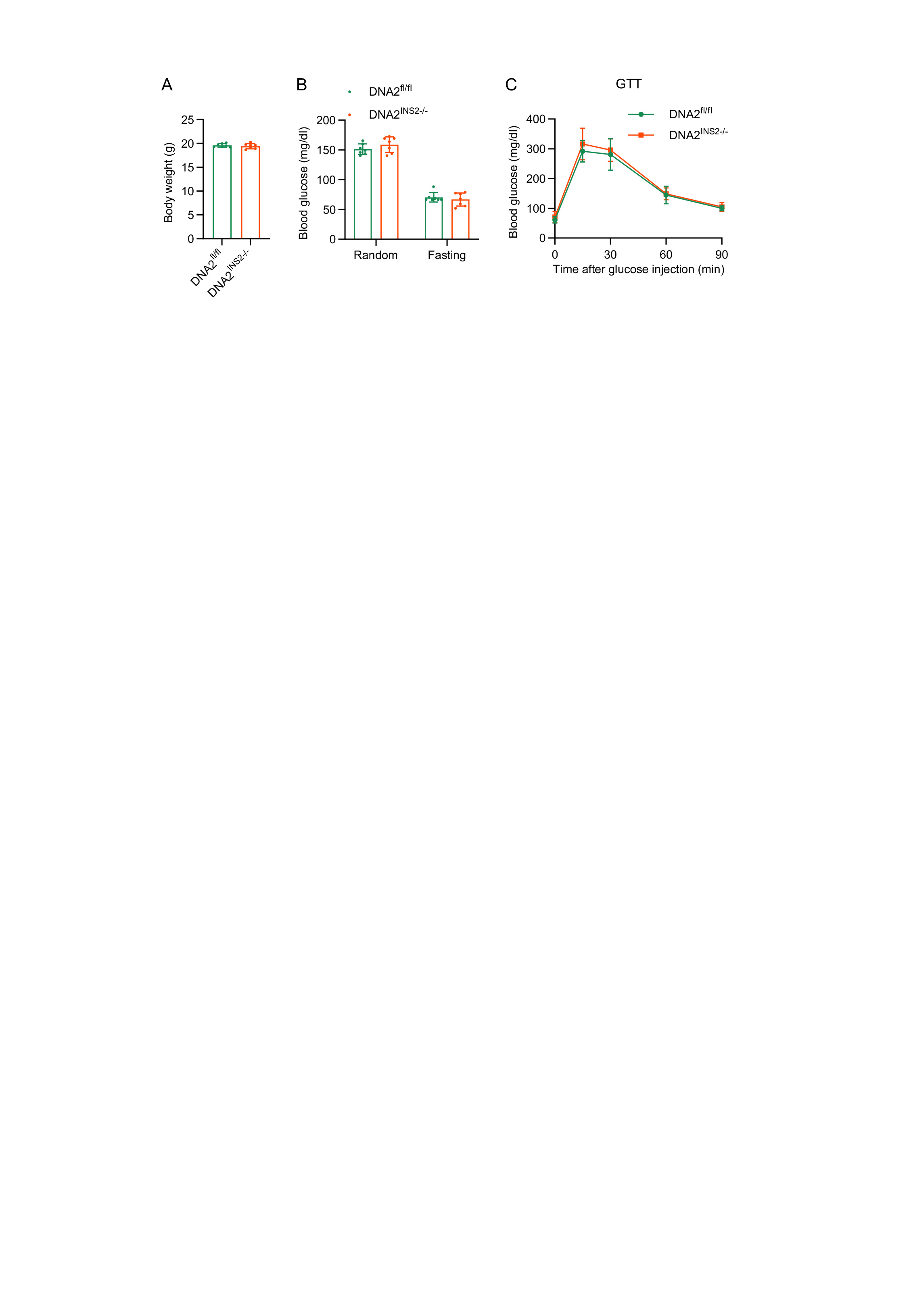

Supplement: Supplementary file 4 [file Image2.jpeg]
