# Supplementary material for: DNA2 acts as a brake on β cell insulin hypersecretion and diet-induced metabolic dysfunction
Source: Front Cell Dev Biol. 2026 Mar 4;14:1733190. doi: 10.3389/fcell.2026.1733190 (PMC12996210; doi:10.3389/fcell.2026.1733190)
Supplement: Supplementary file 2 [file Table1.docx]

Table S1. Primer sequences used for the construction of DNA2 overexpression and point-mutant vectors

| Name | Forward primer (5'-3') | Reverse primer (5'-3') |
| --- | --- | --- |
| Mouse DNA2^WT^ | CTAGCTAGCATGGAAAATTTGAAGCATAT | TATGCTCGAGCTACTTAGAAAAGGTG |
| Mouse DNA2^D278A^ | GGCAAAATAgcaGTCACAGTCGGGGTCAAAATACA | GTGACtgcTATTTTGCCCTTCAGTCCAAACCT |
| Mouse DNA2^K655E^ | GACAGGAgagACAACCACCATCTGTGCCCTGG | TGGTTGTctcTCCTGTCCCTGGCATCCCAACA |

Table S2. Primer sequences used for quantitative real-time PCR (qRT-PCR)

| Name | Forward primer (5'-3') | Reverse primer (5'-3') |
| --- | --- | --- |
| Rat DNA2 | CCGCGGATGGAGATACTTCA | AACGGCTAGCACCAGGTATC |
| Rat β-Actin | AGGCCCCTCTGAACCCTAAG | TCCGGAGTCCATCACAATGC |
| Mouse DNA2 | CAGACCCTCCGAGAAGTGAG | TCCGCCCACTTAGAGAAAGA |
| Mouse Insr | TTCGGGAGAGGATGTGAGAC | CAGCTCATGTAGCCTGGTCA |
| Mouse Akt1 | TTCCTCCTCAAGAACGATGG | GCAGCGGATGATAAAGGTGT |
| Mouse Ngn3 | GTTCCAATTCCACCCCACCT | CGATCATTGGCCTTCTTGCG |
| Mouse ki67 | CCTGTGAGGCTGAGACATGG | TGGCTTGCTTCCATCCTCAT |
| Mouse Igf1r | GCCGACGAGTGGAGAAATCT | AGTGATGACGGTGAGCTTGG |
| Mouse Irs2 | GGCGCAGAACATCCATGAGA | GGTTGACTAGGTGGTGGTGG |
| Mouse Ccnd2 | TACCTCCCGCAGTGTTCCTA | GCCAAGAAACGGTCCAGGTA |
